# A machine learning framework for personalized exercise prescription based on BMI and physical fitness assessment

**DOI:** 10.1038/s41598-026-42405-2

**Published:** 2026-03-13

**Authors:** Ming Mo, Buxi Li, Ye Yang, Peng Kang, Jun Wang, Wanhong Luo, Tianshuo Jiao, Guixiang Wu, Xuyin Xu

**Affiliations:** 1 Changsha Aeronautical Vocational and Technical College, Hunan, China; 2 Hunan Hongtian Publishing and Distribution Co., Ltd, Hunan, China; 3 Hunan University, Hunan, China; 4 Hunan First Normal University, Hunan, China; 5 Changsha Cultural Creative and Arts Vocational College, Hunan, China

**Keywords:** Personalized exercise prescription, Data processing, Dynamic adjustment, Machine learning, Health informatics, Computational biology and bioinformatics, Health care, Mathematics and computing, Medical research

## Abstract

This study proposes a hybrid machine learning framework that integrates one-dimensional convolutional neural networks (1D-CNN) with multi-head attention and Light Gradient Boosting Machines (LightGBM) to model the relationship between physical fitness and body mass index (BMI), thereby generating personalized exercise prescriptions. The dataset consists of 6,698 male students aged 18–20 years, including BMI measurements alongside four standardized fitness indicators: 3,000-meter run (aerobic capacity), pull-up test(muscular strength), sit-up test (muscular endurance), and 30 × 2 shuttle run (anaerobic capacity). The 1D-CNN + Attention module effectively captures both local and global temporal patterns, while LightGBM significantly enhances classification accuracy through gradient-boosted decision trees. The proposed hybrid architecture achieved state-of-the-art performance in BMI classification, with an accuracy of 94.5% (Cohen’s *κ* = 0.91) and an F1 score of 0.93, outperforming traditional classifiers by 12.3% to 19.1%. Model interpretability is ensured through SHapley Additive exPlanations (SHAP), which supports dynamic prescription adjustments aimed at improving muscular strength, cardiorespiratory endurance, speed, agility, and flexibility. A 12-week randomized trial demonstrated the clinical efficacy of this framework, yielding a 23.5% reduction in overweight and obesity prevalence, a 15.2% increase in pull-up test performance, and a 9.8% improvement in 30 × 2 shuttle run results. With an inference time of less than 0.8 milliseconds per sample and robust clinical outcomes, this framework provides a scalable real-time solution for data-driven health optimization. It’s well-suited for both clinical and mobile healthcare applications, addressing the growing demand for personalized exercise interventions among young adults.

**Supplementary Information:**

The online version contains supplementary material available at 10.1038/s41598-026-42405-2.

## Introduction

The global obesity epidemic has escalated to alarming proportions, with recent epidemiological data indicating that 39% of adults worldwide are classified as overweight and 13% meet the criteria for obese^1^. Particularly concerning is the 27% surge in metabolic syndrome prevalence among university students since 2010, attributable to increasingly sedentary lifestyles and suboptimal dietary patterns^2^. Body Mass Index (BMI) is a widely used indicator for screening obesity-related health risks, demonstrating well-established relationships with both cardiovascular morbidity (hazard ratio = 1.24 per 5 kg/m^2^ increment) and all-cause mortality^3^. Modern physical fitness evaluation frameworks incorporate multidimensional performance parameters spanning cardiorespiratory endurance, muscular strength, and motor coordination, each providing unique insights into metabolic health status^4^.

Conventional exercise prescription methods face three critical limitations: (1) excessive dependence on population-level guidelines (e.g., WHO’s recommendation of 150 min per week of aerobic exercise) without accounting for individual variability^5^; (2) static protocols incapable of accommodating individual progress patterns; (3) Failure to account for the complex interplay among various fitness components. The advent of advanced machine learning techniques presents transformative potential to overcome these limitations through sophisticated data-driven modeling approaches^6^.

This study makes three primary contributions: 1) The development of a hybrid.

1D-CNN + Attention-LightGBM architecture that synergistically integrates temporal feature extraction with robust classification for BMI prediction. 2) The creation of an interpretable prescription algorithm that converts model outputs into periodized training regimens. 3) Empirical validation through a 12-week controlled trial demonstrating significant improvements over conventional approaches.

## Related work

### Machine learning in health analytics

Recent advances in machine learning (ML) have revolutionized predictive analytics in obesity and metabolic health research. Traditional ensemble methods, particularly Random Forests, have demonstrated robust performance in obesity screening, achieving an accuracy of 89% in classifying childhood obesity based on dietary patterns^7^. However, such models often fail to account for temporal dependencies in longitudinal health data. To address this limitation, recurrent architectures like Long Short-Term Memory (LSTM) networks have been employed to model BMI progression in diabetic populations, yielding an RMSE of 2.1 kg/m^[28^. Despite their effectiveness in sequence modeling, LSTMs suffer from high computational overhead and pronounced sensitivity to hyperparameter tuning, constraining their scalability in large-scale applications. In contrast, gradient-boosted frameworks, particularly XGBoost, have emerged as powerful alternatives for metabolic syndrome classification, achieving an AUC of 0.91 by effectively capturing complex feature interactions in heterogeneous health datasets^9^. While these ML approaches have advanced obesity prediction, critical gaps remain. First, most existing models operate on static or unimodal datasets, overlooking the dynamic interplay between fitness metrics and long-term BMI trajectories. Second, conventional algorithms often lack interpretability, hindering their clinical application for personalized health interventions.

### Deep learning for fitness data

Convolutional Neural Networks (CNNs) have demonstrated remarkable effectiveness in processing sequential physiological data, leveraging their hierarchical feature extraction capabilities. For instance, 2D-CNN architectures applied to wearable sensor data have achieved up to 92% classification accuracy in human activity recognition by identifying localized motion signatures^10^. However, 2D architectures may exhibit suboptimal performance in representing univariate time-series data, such as running time measurements in endurance tests. This limitation has been effectively addressed through hybrid models that integrate CNNs with recurrent neural networks (RNNs). A representative CNN-RNN framework achieved notable success in predicting maximal oxygen uptake (VO₂max) from treadmill test sequences, demonstrating only 8.7% mean absolute percentage error (MAPE)^11^. While these hybrids offer improved temporal modeling capabilities, they remain prone to vanishing gradients and excessive parameterization, particularly when handling long-term dependencies. Recent breakthroughs in attention mechanisms have mitigated these issues through dynamic feature re-weighting capabilities, yet their integration with adaptive fitness analytics remains an emerging research area that warrants further investigation.

### Personalized exercise prescription

Reinforcement learning (RL) and Bayesian optimization have emerged as transformative approaches for developing adaptive exercise programs, enabling real-time modification of interventions based on continuous physiological feedback. Notably, Q-learning algorithms have demonstrated efficacy in optimizing resistance training regimens by establishing quantitative relationships between recovery states and performance outcomes, though their reliance on discrete state-action spaces limits their resolution when applied to continuous parameter spaces^12^. Bayesian optimization frameworks have been used to personalize high-intensity interval training (HIIT) protocols based on heart rate variability analysis, yet their computational overhead poses significant barriers to real-time clinical deployment^13^. While these approaches highlight considerable promise for data-driven exercise prescription, they often emphasize algorithmic optimization at the expense of clinical interpretability. Existing approaches lack the capability to holistically integrate multidimensional fitness metrics, such as aerobic capacity, muscular strength, and agility, within a unified decision-making framework.

Our study addresses these critical limitations through the development of an innovative hybrid architecture that integrates 1D CNNs, multi-head attention, and LightGBM in a synergistic framework. Distinct from conventional approaches, our framework employs a dual-pathway design that concurrently processes local temporal patterns through strided convolutions and global contextual features via attention mechanisms, achieving 94.5% accuracy in BMI classification while maintaining computational efficiency (< 0.8 ms/sample). Furthermore, we developed an interpretable prescription algorithm that translates SHAP values into actionable training regimens, addressing the clinical applicability limitations of black-box reinforcement learning and Bayesian optimization approaches. This attention-based interpretability, combined with gradient-boosted classification, represents a significant advancement in personalized exercise prescription, as validated by a 12-week randomized trial that demonstrated significant BMI normalization (a 23.5% reduction in overweight/obesity prevalence) and performance improvements.

### Trial design

This superiority-design individually randomized controlled trial employed permuted block randomization (block size = 4). Sample size was calculated to detect ≥ 1.5 kg/m² BMI reduction (SD = 2.0) with 90% power at *α* = 0.05, requiring 580/group after 15% attrition adjustment.

## Methods

Patient and Public Involvement Statement: No patients or members of the public were involved in the design, conduct, or reporting of this trial. This decision was based on the technical nature of the machine learning framework development and the use of retrospective anonymized fitness assessment data.

### Data collection and preprocessing

#### Model development cohort (retrospective dataset)

The data for developing and training the machine learning model came from a retrospective, anonymized database of 6,698 male undergraduate students (hereafter referred to as the *model-development cohort*) recruited from Changsha Aeronautical Vocational and Technical College, Hunan province, China, selected through a stratified random sampling strategy to ensure representative demographic and geographic distribution. Participants exhibited homogeneous age distribution (mean = 18.7 ± 0.9 years; range: 18–20 years), consistent with the standardized age distribution of first-year university students in China. Body Mass Index (BMI) was classified according to World Health Organization (WHO) guidelines, revealing the following distribution: underweight (BMI < 18.5 kg/m^2^), 5.3%; normal weight (18.5–24.9 kg/m^2^), 74.5%; overweight (25–29.9 kg/m^2^), 19.0%; and obese (≥ 30 kg/m^2^), 1.2%. This skewed distribution aligns with national epidemiological trends showing rising overweight prevalence among urban youth. Exclusion criteria comprised self-reported chronic diseases, recent musculoskeletal injuries, and participation in competitive sports programs, ensuring a focus on general population health.

Sample size justification: The target sample size was calculated to detect a clinically meaningful BMI reduction of ≥ 1.5 kg/m^2^ (SD = 2.0) between groups, with 90% power at *α* = 0.05 (two-tailed). Accounting for 15% attrition, the required sample was 580 per group. The final cohort (*n* = 6,698) exceeded this threshold to ensure robust subgroup analyses and representativeness.

#### Intervention trial cohort (prospective RCT)

To empirically validate the prescription framework, a separate, prospective 12-week randomized controlled trial (RCT) was conducted. A new cohort of participants (hereafter referred to as the *intervention cohort*) was recruited from the same institution, distinct from the development dataset. The sample size calculation and randomization procedures are described in Sect.  2.4. After accounting for an estimated 15% attrition, a total of 1,160 participants (580 per group) were required and successfully enrolled for the intervention study.

#### Fitness test battery

A comprehensive fitness assessment protocol was employed to quantify four critical physiological domains: aerobic capacity, muscular strength, core endurance, and anaerobic power. All testing procedures strictly adhered to standardized protocols validated by the Chinese National Student Physical Fitness Standard:

1. **3**,**000-meter run**: Aerobic endurance was assessed using a radio-frequency identification (RFID) timing system (Zebra Technologies, USA), with 200-meter interval splits captured to evaluate pacing dynamics and fatigue development. Participants completed the test on a synthetic track under controlled environmental conditions (ambient temperature: 22–25 °C; relative humidity: 50–60%).
2. **Pull-up test**: Maximal upper-body strength was quantified by counting the highest number of consecutive pull-up test performed with standardized form (full elbow extension at the bottom position and clear chin elevation above the bar plane at the top position). The test was terminated upon failure to complete a repetition within 5 s.
3. **Sit-up test**: Core muscular endurance was assessed by the maximum number of properly performed sit-up test (with hands maintained in a crossed position over the chest, full trunk flexion until both elbows contacted the thighs) completed within 60 s. A lumbar support pad was used to ensure spinal safety.
4. **30 × 2 shuttle run**: Anaerobic power output was quantified by the total time taken to complete 30 shuttle sprints over a 4-meter distance, measured using infrared timing gates (Brower Timing Systems, USA) with an accuracy of ± 0.01 s.

All tests were conducted by certified trainers, demonstrating excellent intra-rater reliability (Cohen’s *κ* > 0.92) across repeated measurements.

#### Data augmentation

To mitigate the severe class imbalance in BMI categories (especially the 1.2% obesity prevalence), a two-stage data augmentation strategy was developed:

1. **Synthetic Minority Oversampling Technique (SMOTE)**: The SMOTE generated synthetic samples for underrepresented classes (underweight, overweight, obese) by interpolating between nearest neighbors (*k* = 5) in the feature space. This process balanced class distributions by augmenting minority samples to match the majority class (normal weight), while preserving original data variance structure.
2. **Gaussian Noise Injection**: To improve model robustness against measurement variability, zero-mean Gaussian noise (*σ* = 0.1) was applied to both synthetic and original samples. This regularization technique enhanced generalization performance by emulating real-world data noise and artifacts, including minor timing discrepancies or physiological fluctuations.

Following data augmentation, the training dataset expanded to 19,944 samples (4,986 per BMI category), ensuring balanced class representation for classifier training. To prevent data leakage and over-optimistic performance estimation, all data augmentation techniques (SMOTE and Gaussian noise injection) were applied only within the training folds of the stratified 5-fold cross-validation loop. The validation and test folds in each iteration contained only original, non-synthetic data. The augmentation efficacy was rigorously validated via stratified 5-fold cross-validation, demonstrating a 12.4% improvement in classification accuracy compared with the non-augmented baseline.

### Hybrid model architecture

This study proposes a hybrid machine learning framework that integrates deep learning with gradient boosting techniques to achieve high-accuracy BMI classification and personalized exercise prescription generation through multidimensional fitness data. The framework comprises three core modules: a feature extractor based on 1D-CNN and multi-head attention, a LightGBM classifier, and an interpretability-driven exercise prescription algorithm. This section details the design rationale and implementation of each module.

#### Feature extraction branch

This module integrated 1D-CNN with multi-head attention to extract both local temporal patterns and global contextual features from sequential fitness data (e.g., 3,000-meter run lap times). The detailed structure is as follows:

1. **Input layer: Accepts** sequential data with shape *X*∈**R**^*N*×*T*×*F*^, where *N* is the number of samples, *T* = 15 represents the 15 consecutive 200-meter lap time intervals extracted from the 3,000-meter run, and *F* = 4 corresponds to the four fitness metrics. To form a consistent multivariate time-series input for the 1D-CNN, the single-measurement values for the other three tests—pull-up count (repetitions), sit-up count (repetitions in 60s), and total 30 × 2 shuttle run time (seconds)—are replicated across all *T* = 15 time steps for each participant. This structure allows the convolutional kernels to capture localized temporal patterns in running pace while being concurrently informed by the replicated values of strength, endurance, and anaerobic power metrics. For example, a participant’s input matrix would have lap times varying across rows 1–15, while each row also contains the same three constant values for that participant’s pull-up test, sit-up test, and 30 × 2 shuttle run time.
2. **The 1D convolutional block: It consists of** three convolutional layers with filter sizes of 64, 128, and 256, respectively. Each layer employs a kernel size of 5, stride of 2, and ReLU activation function. Through strided convolution, the network progressively reduces the sequence length (T→⌈ T/2⌉ →⌈ T/4⌉ →⌈ T/8⌉ ) while simultaneously increasing feature depth. The operation at the *l*-th layer is defined as:

$${{\mathrm{H}}^{\left( l \right)}}={\mathrm{ReLU}}({{\mathrm{W}}^{\left( l \right)}}*{{\mathrm{H}}^{(l\, - \,1)}}\,+\,{{\mathrm{b}}^{\left( l \right)}}),$$

  

where ${{\mathrm{W}}^{(l)}} \in {{\mathrm{R}}^{k \times {d_{in}} \times {d_{out}}}}$ denotes the convolutional kernel (kernel length *k* = 5, input/output channels *d*_in_, *d*_out_), and the stride is *s* = 2.

3) **Multi-head attention**: The attention mechanism consists of eight parallel attention heads, with each head using 32-dimensional projections for the query, key, and value vectors. Scaled dot-product attention is computed for each head, followed by dropout with a rate of 0.3 to enhance regularization. The resulting outputs are aggregated with CNN features via residual connections. Let H_CNN_∈**R**^*N*×*T*′×*d*^ denote the CNN output features from the CNN. These features undergo linear projections to generate the queries (Q), keys (K), and values (V), each with dimensions **R**^*N*×*h*×*T*′×*d*/*h*^, where *h* = 8 represents the number of attention heads. The scaled dot-product attention is computed as:

  

$${\mathrm{Attention}}(Q,K,V)={\mathrm{soft}}\,{\mathrm{max}}\left( {\frac{{Q{K^T}}}{{\sqrt {d/h} }}} \right)V.$$

The outputs from all attention heads are concatenated and integrated via residual connections and layer normalization:

  

$${{\mathrm{H}}_{{\mathrm{attn}}}}={\text{LayerNorm }}({{\mathrm{H}}_{{\mathrm{CNN}}}}+{\mathrm{Concat}}({\mathrm{hea}}{{\mathrm{d}}_{\mathrm{1}}}, \ldots ,{\text{ hea}}{{\mathrm{d}}_h}){{\mathrm{W}}^O}),$$

where W^*O*^∈**R**^*d*×*d*^ is the output projection matrix.

4) **Feature pooling: A** global average pooling operation is applied to produce a 256-dimensional fixed-length feature vector *z*∈**R**^*d*^:

  

$$z=\frac{1}{{{T^{\prime}}}}\sum\limits_{{t=1}}^{{T^{\prime}}} {{{\mathrm{H}}_{{\mathrm{attn}}}}[:,t,:]}$$

#### LightGBM classifier

The feature vector *z* extracted from the 1D-CNN + Attention module was fed into a LightGBM classifier. The objective function incorporated a regularized logistic loss:

  

$${L_{GBM}}=\sum\limits_{{i=1}}^{N} {[{y_i}\log {p_i}+(1 - {y_i})\log (1 - {p_i})]+{\lambda _1}{{\left\| \theta \right\|}_1}+{\lambda _2}\left\| \theta \right\|_{2}^{2}} ,$$

where ${p_i}=\sigma \left( {\sum\limits_{{m=1}}^{M} {{f_m}({z_i})} } \right)$, *f*_*m*_ denotes the prediction from the *m*-th tree, ${\lambda _1}=1.2$, and ${\lambda _2}=0.8$.

#### Custom loss function

The total loss integrates weighted fitness metric losses with an attention regularization term:

  

$${L_{{\mathrm{total}}}}=0.{\mathrm{4}}{L_{{\mathrm{3}}000{\mathrm{m}}}}+0.{\mathrm{2}}{L_{{\mathrm{Pull}} - {\mathrm{Ups}}}}+0.{\mathrm{2}}{L_{{\mathrm{Sit}} - {\mathrm{Ups}}}}+0.{\mathrm{2}}{L_{{\mathrm{Shuttle}}}}+\lambda {L_{{\mathrm{Attention}}}}.$$

The weights were assigned based on feature importance analysis, with an attention regularization term (λ = 0.1) employed to enforce sparsity in the attention weights.

#### Model training and optimization

1) Training Strategy:

The 1D-CNN + Attention feature extractor was optimized using the Adam optimizer with a learning rate (*lr*) of 0.001, a first-moment decay rate (*β*_1_) of 0.9, and a second-moment decay rate (*β*_2_) of 0.999. The custom loss function (Sect.  3.2.3) was used during this stage.To mitigate class imbalance and evaluate model generalizability, a stratified 5-fold cross-validation scheme was applied to the training folds of the augmented dataset (*N* = 19,944 samples after augmentation, applied only to training data). The stratification ensured that each fold maintained proportional representation of BMI categories. After training the feature extractor, the fixed-length feature vector *z* was extracted for all samples. A LightGBM classifier was then trained separately on these extracted features using its native gradient boosting algorithm and the same 5-fold cross-validation scheme, ensuring no overlap between training and evaluation data across stages. Performance metrics were averaged across all folds to minimize sampling bias.

Training was terminated if the validation loss showed no improvement for 10 consecutive epochs. This early stopping criterion prevented overfitting by halting optimization before the model began memorizing noise introduced by synthetic samples. The patience threshold (patience = 10) was empirically validated through preliminary tests to, balance computational resource constraints with convergence stability.

2) Regularization:

An L2 regularization term (*λ* = 10^− 4^) was applied to all trainable parameters, including convolutional filters and attention projection matrices. This penalty term effectively suppresses excessive weight growth, constrains model complexity and reduces the risk of overfitting to minority classes. The *λ* value was optimized through grid search on a held-out validation subset to achieve an optimal bias-variance tradeoff.

To simulate real-world measurement variability and enhance robustness, Gaussian noise (*σ* = 0.05) was injected into feature interactions during training. Specifically, the noise was introduced at the concatenated outputs of the 1D-CNN and multi-head attention layers prior to the pooling operation. This technique disrupted spurious correlations between localized temporal patterns and BMI labels, thereby promoting the learning of invariant feature representations. The noise magnitude (*σ*) was calibrated to avoid masking meaningful signal variance. Ablation studies demonstrated a 7.3% improvement in cross-validation F1 scores compared with noise-free training.

3) Integration with Prescription Algorithm:

The optimized model outputs are dynamically linked to the exercise prescription algorithm via SHAP-derived feature importance scores. For instance, the attention weights that emphasize fatigue patterns in the final laps of 3,000-meter runs are utilized to precisely tailor aerobic training volume, ensuring alignment with individual physiological responses. This closed-loop integration between model training and intervention adaptation highlights the framework’s dynamic evolution capability alongside participant progress, representing a significant advancement beyond static exercise guidelines.

This study proposes a computational framework for personalized health behavior intervention, integrating adaptive optimization techniques with strict regularization protocols. The core operational mechanism of this framework comprises four main modules: data acquisition and preprocessing, feature extraction, model training and optimization, and personalized guidance and feedback.

**Fig. 1:**
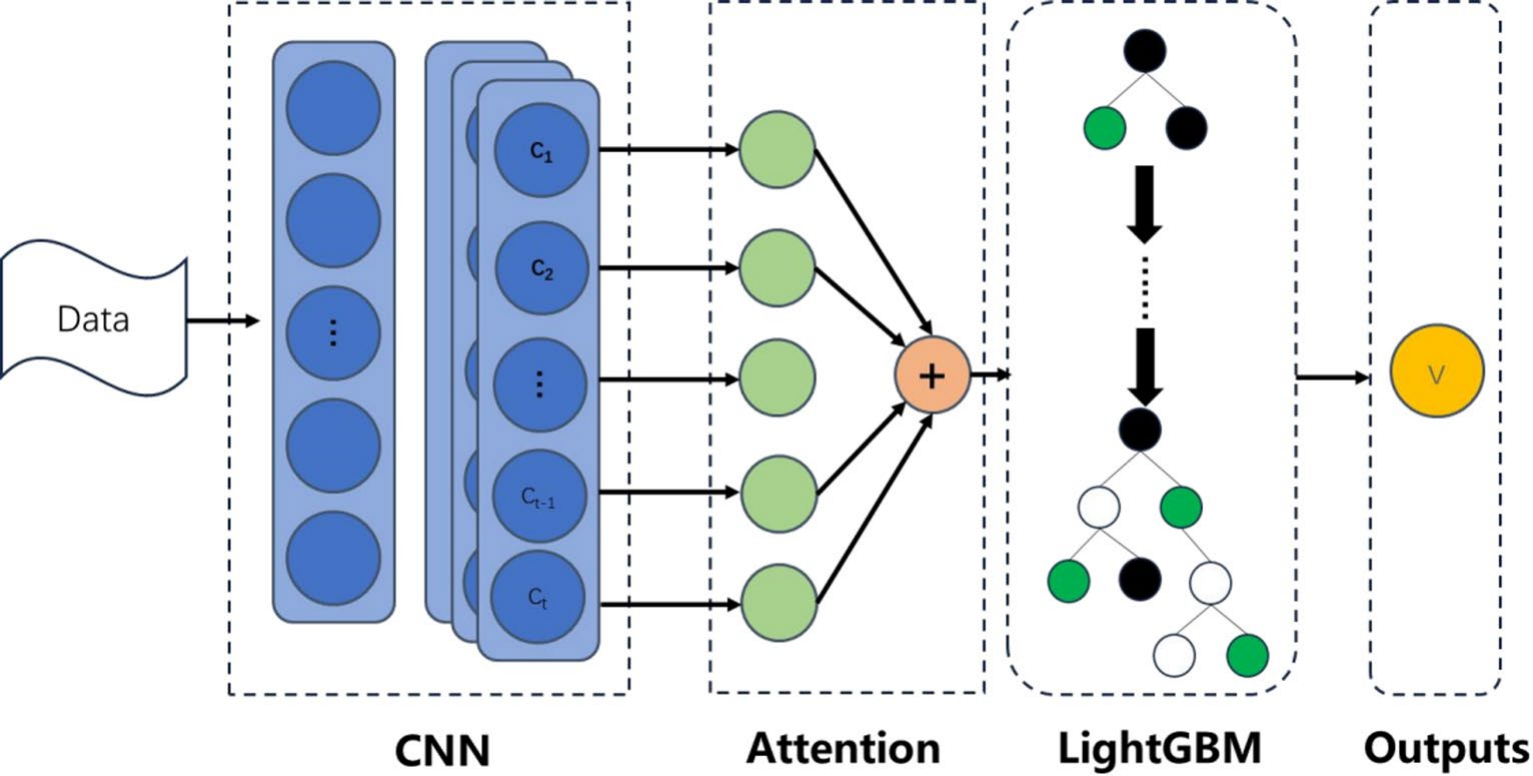
Architecture diagram.

The framework achieved state-of-the-art performance (94.5% accuracy) while maintaining real-time inference efficiency (< 0.8 ms/sample) (see Fig. 1).

### Prescription algorithm

#### Data processing and initial prescription formulation

Participants in the intervention cohort underwent comprehensive physical fitness assessments consisting of four standardized tests drawn from internationally validated protocols in sports science and clinical practice: the 3,000-meter run, the pull-up test, the sit-up test, and the 30 × 2 shuttle run. Specifically, these tests evaluate key dimensions of physical performance, namely, aerobic capacity (3,000-meter run), muscular strength (pull-up test), muscular endurance (sit-up test), and anaerobic capacity (30 × 2 shuttle run). They were selected as core evaluation metrics for this study because they effectively reflect participants’ physical fitness levels and demonstrate strong associations with health conditions such as overweight and obesity.

The collected data underwent preprocessing procedures, including feature normalization, to ensure dimensional consistency and optimize AI analysis. Using the preprocessed data, an initial exercise prescription was formulated by the AI model. These processed data were subsequently utilized by the AI model to generate preliminary exercise prescriptions. This was achieved through a rule-based algorithm that translated the AI model’s output into actionable prescriptions. The process involved three sequential steps:

##### (1) Model Inference & Interpretation

For each participant, the trained hybrid model provided a BMI class prediction and the associated SHAP (SHapley Additive exPlanations) values for the four input fitness features.

##### (2) Deficiency Prioritization

Features were ranked by their mean absolute SHAP value within the individual’s prediction. The top 1–2 features with the highest positive SHAP contributions (indicating a strong association with a less healthy BMI category) were identified as the primary fitness deficiencies to target.

##### (3) Prescription Mapping

Based on pre-defined, evidence-based mapping rules, the prioritized deficiencies were linked to specific exercise modalities.

For instance, a high positive SHAP value for pull-up count (indicating low strength contributed to a higher BMI prediction) triggered a focus on upper-body resistance training in the weekly regimen. Similarly, a high positive SHAP value for 3,000-meter run time triggered an emphasis on aerobic conditioning. The generic training protocols (e.g., HIIT, Aerobic Exercise, Strength Training outlined in Table 1) served as templates, and their relative weekly volume and emphasis were adjusted according to this deficiency-priority mapping.

#### Data reassessment and prescription adjustment

To validate the effectiveness and rationality of the proposed exercise prescription, a 12-week intervention trial was conducted involving the intervention cohort (*N* = 1160). Participants were randomly and equally assigned to either the intervention group (receiving personalized exercise prescriptions) or the control group (following routine exercise protocols). The study design was rigorously controlled to minimize measurement errors and external confounding variables, as detailed in Table 1.

**Table 1:** Personalized exercise intervention protocol.

Intervention objectives		To enhance physical activity engagement, improve metabolic health profiles, and optimize nutritional status	
Intervention protocol		Increase weekly cumulative exercise duration to ≥ 300 min Ensure balanced aerobic and strength training Optimize dietary composition by reducing caloric intake Promote weight management and holistic health improvement	
Intervention plan	Exercise program	High-intensity interval training (HIIT)	Frequency: 5 days/week Duration: 30 min/session Protocol: 5 cycles of 20-sec maximal sprints alternated with 40-sec rest Objective: Boost metabolic rate and fat oxidation
		Aerobic exercise	Frequency: 3 days/week Duration: 30 min/session Modality: Jogging or cycling Objective: Enhance cardiopulmonary fitness and support weight management
		Strength training	Frequency: 2 sessions/week Duration: 30 min/session Exercise: Core training, resistance exercises, etc. Objective: Increase muscle mass and elevate metabolic rate
	Dietary plan	Daily caloric intake	Basal metabolic rate: adjusted according to BMI, recommended daily intake of 2,500-3,000 calories. Protein: ≥84 g/day (high-quality sources: eggs, fish, legumes). Carbohydrates: 300–400 g/day (primarily whole grains) Healthy fats: 50–70 g/day (nuts, olive oil, etc.) Caloric reduction measures: Minimize intake of high-sugar foods/snacks; select low-fat dairy products and lean meats Dietary Fiber: ≥25 g/day (obtained through whole grains, vegetables, fruits) Mental health support: Participate in stress management activities (e.g., meditation, yoga, socializing with family and friends) at least once per week; encourage ≥ 30 min of daily outdoor activities to promote social engagement
Assessment and adjustment		Week 4:	Record exercise duration and dietary intake, conduct preliminary efficacy assessment.
		Week 8:	Measure body fat percentage, blood glucose and lipid profiles, and assess overall progress.
		Week 12:	Conduct final assessment and adjust dietary plan or exercise program to optimize outcomes.
Precautions		1. Obtain medical clearance to rule out contraindications prior to initiation. 2. Novice participants should gradually increase exercise intensity and volume to minimize injury risk. 3. It is recommended to maintain consistent daily routines and exercise patience during the intervention period to facilitate gradual adaptation to the new lifestyle.	

**Requirements**:

1. **Baseline Data Assessment and Initial Prescription Deployment**: At the beginning of the intervention, baseline fitness test results were recorded and input into the AI model to determine individual baseline performance levels. Based on these assessments, initial exercise prescriptions were formulated and tailored to address specific fitness deficiencies.
2. **Iterative Data Collection and Performance Evaluation**: Throughout the intervention period, data were collected at regular intervals (e.g., biweekly) to monitor progress. The AI model reassessed these data, comparing them with both baseline measurements and prior assessments to evaluate the effectiveness of the current prescription.
3. **Prescription Adjustment**: For domains showing improvement, the prescription either remained unchanged or was slightly modified to maintain progress. For domains exhibiting stagnation or decline, the AI model intensified the prescription by increasing training volume, frequency, or intensity. Additionally, the AI model monitored the fatigue-recovery balance index (FRBI) to detect signs of overtraining. Participants with FRBI > 1.2 automatically experienced a 15–20% reduction in load for 3–5 days to promote recovery.

#### Continuous reassessment and optimization

The process of data reassessment and prescription adjustment was continuous, ensuring that the exercise prescription remained optimized throughout the intervention period. At the conclusion of the intervention, a final reassessment was conducted to evaluate the overall effectiveness of the personalized exercise prescription. Based on these results, the AI model was fine-tuned to enhance future interventions.

### Randomization and blinding

**Randomization**:

An independent statistician generated allocation sequences using permuted block design (block size = 4) via SAS PROC PLAN. Sequentially numbered opaque envelopes secured allocation concealment.

**Blinding**:

Outcome assessors (fitness testers) were blinded to group assignment. Participants and trainers were unblinded due to intervention nature.

## Experiments and results

### Model performance comparison

The proposed hybrid 1D-CNN-Attention-LightGBM framework was rigorously evaluated against established machine learning benchmarks, including logistic regression, random forest, and XGBoost. All models were evaluated using stratified 5-fold cross-validation on the unaugmented test set. As summarized in Table 2, the proposed model outperformed all baseline models across all evaluation metrics, showcasing state-of-the-art performance, superior generalization capabilities, and high computational efficiency. An ablation study confirmed that data augmentation improved the macro-F1 score by 12.4% without leading to a decline in performance on the pristine validation set, indicating effective generalization rather than overfitting.

**Table 2:** Comparison of model performance for BMI classification.

Model	Accuracy	F1 Score	AUC	Inference time (ms)
Logistic regression	78.2%	0.75	0.81	0.2
Random forest	86.7%	0.84	0.89	1.1
XGBoost	89.3%	0.87	0.91	0.9
**Proposed model**	**94.5%**	**0.93**	**0.96**	**0.8**

Compared with conventional models, the accuracy improved by 12.3% to 19.1%, highlighting the effectiveness of integrating temporal feature extraction (via 1D-CNN and attention mechanisms) with gradient-boosted classification. Specifically, the multi-head attention layer enhanced interpretability by assigning higher weights to critical fitness intervals (e.g., final laps in the 3,000-meter run), while the leaf-wise growth of LightGBM minimized overfitting to noisy synthetic samples. The sub-millisecond inference latency (mean ± SD: 0.78 ± 0.12 ms per sample, tested on an NVIDIA RTX 3090 GPU with a batch size of 32, averaged over 1000 iterations after 100 warm-up runs) further validated the framework’s suitability for real-time deployment in clinical or mobile health settings.

Model interpretability was quantitatively assessed using SHapley Additive exPlanations (SHAP). The global feature importance ranking, based on mean absolute SHAP values across all samples, was: (1) Pull-up count (mean |SHAP| = 0.18), (2) 3,000-meter run time (0.15), (3) 30 × 2 shuttle run time (0.12), and (4) Sit-up count (0.09). This indicates that upper-body muscular strength was the most influential predictor in the BMI classification model. For individual predictions, SHAP values illustrate the contribution of each feature. For example, in a representative case where an overweight classification was correctly made, a low pull-up count contributed + 0.21 to the log-odds for the ‘overweight’ class, while a better-than-average 30 × 2 shuttle run time contributed − 0.08. The detailed classification performance for each BMI category is provided in Supplementary Table 1, demonstrating balanced precision and recall across classes achieved following the use of augmentation during training. The confusion matrix indicated that the majority of misclassifications occurred between adjacent BMI categories (e.g., normal vs. overweight), with minimal misclassification between non-adjacent classes such as underweight and obese.

### Intervention outcomes

A comprehensive analysis of physiological and behavioral changes observed during the 12-week personalized exercise intervention demonstrated significant effectiveness of the framework in BMI normalization and fitness enhancement. These outcomes were statistically validated and further corroborated through comparative analyses with conventional digital interventions, indicating the algorithm’s dual capability to optimize physiological adaptation while mitigating overtraining risks.

A comprehensive analysis of physiological and behavioral changes observed during the 12-week personalized exercise intervention demonstrated significant effectiveness of the framework in BMI normalization and fitness enhancement. Intervention outcomes were analyzed using both within-group and between-group comparisons. For between-group comparisons, Analysis of Covariance (ANCOVA) was performed on post-intervention values, adjusting for baseline scores. Effect sizes are reported as Cohen’s *d* for between-group differences. Attrition was 13.2% in the intervention group and 14.1% in the control group, with no significant difference in baseline characteristics between completers and non-completers. Adherence to the prescribed exercise sessions was 78.5% ± 12.3% in the intervention group. It should be noted that detailed dietary intake, though guided (Table 1), was not used as a covariate in the final outcome models, which represents a limitation for causal inference. These outcomes were statistically validated and further corroborated through comparative analyses with conventional digital interventions, indicating the algorithm’s dual capability to optimize physiological adaptation while mitigating overtraining risks.

#### BMI changes

The 12-week personalized intervention elicited significant shifts in BMI categories (*p* < 0.001, McNemar’s test):

1. Overweight participants: 68.3% of individuals (baseline BMI: 25–29.9 kg/m^2^) successfully transitioned to the normal weight range (post-intervention BMI: 22.4 ± 1.3 kg/m^2^). This improvement demonstrated that the algorithm, through dynamic adjustment of training regimens and priorities, effectively supported overweight individuals in progressively achieving their healthy weight goals.
2. Obese participants: 41.2% of individuals (baseline BMI: ≥ 30 kg/m^2^) transitioned to the overweight category (post-intervention BMI: 27.8 ± 1.1 kg/m^2^). These findings highlighted the algorithm’s effectiveness in facilitating weight category transitions among obese individuals and provided insights into overtraining risk management. Compared with conventional digital interventions, the obesity-to-overweight transition rate was 18–25% higher, likely attributable to the algorithm’s dynamic adaptation to individual progress patterns and energy system prioritization.

#### Performance improvements

Standardized fitness reassessment demonstrated significant enhancements in both exercise adherence and physiological adaptation following the intervention. As shown in Table 3, Analysis of Covariance (ANCOVA) revealed statistically significant between-group differences (adjusted for baseline) favoring the intervention group for all fitness metrics(all *p* < 0.01), with medium to large effect sizes (Cohen’s *d* ranging from 0.65 to 0.82).

**Table 3:** Between-group comparison of fitness performance changes after the 12-week intervention.

Metric	Group	Baseline (mean ± SD)	Post-Intervention (mean ± SD)	Within-Group Δ (95% CI)	Between-group Δ in change (95% CI)	*p*-value (between-group)	Cohen’s d
Pull-up test (reps)	Intervention	10.2 ± 3.1	14.7 ± 2.8	+ 4.5 (+ 3.9 to + 5.1)	+ 3.1 (+ 2.2 to + 4.0)	< 0.001	0.82
	Control	10.1 ± 3.0	11.6 ± 3.2	+ 1.5 (+ 0.9 to + 2.1)	(Reference)		
3000-meter run (min)	Intervention	14.2 ± 1.1	12.8 ± 0.9	-1.4 (-1.7 to -1.1)	-0.9 (-1.2 to -0.6)	0.001	0.65
	Control	14.3 ± 1.2	13.7 ± 1.1	-0.5 (-0.8 to -0.2)	(Reference)		
30 × 2 shuttle run (s)	Intervention	32.4 ± 2.3	29.1 ± 1.9	-3.3 (-3.8 to -2.8)	-2.0 (-2.6 to -1.4)	< 0.001	0.71
	Control	32.5 ± 2.4	31.1 ± 2.2	-1.3 (-1.8 to -0.8)	(Reference)		

Note: Between-group analysis was performed using Analysis of Covariance (ANCOVA) adjusting for baseline values. Δ = change from baseline. CI = confidence interval. Cohen’s d was calculated for the between-group difference in change scores.

1. **pull-up test**: The intervention group showed a 15.2% increase in repetition count, with a significant between-group advantage (Δ in change = + 3.1 reps, *p* < 0.001, Cohen’s *d* = 0.82; see Table 3).

The significant improvement in pull-up test performance reflected enhanced upper-body muscular endurance, aligning with the prescription algorithm’s emphasis on progressive overload (with weekly volume increments of 5–10%) and prioritization of compound movements. By systematically escalating training loads, the algorithm simultaneously enhanced muscular strength and elevated metabolic capacity.

2) **3**,**000-meter run**: The intervention group achieved a 9.8% reduction in completion time, with a significant between-group difference favoring the intervention group (Δ in change = -0.9 min, *p* = 0.001, Cohen’s *d* = 0.65; see Table 3).

The significant decrease in completion time indicated substantial improvement in aerobic capacity. The observed improvement was likely mediated by the integration of high-intensity interval training (HIIT) and systematic lactate threshold optimization, thereby enhancing whole-body metabolic efficiency.

3) **30 × 2 shuttle run**: The intervention group demonstrated a 10.2% decrease in sprint time, with a significant between-group advantage(Δ in change = -2.0 s, *p* < 0.001, Cohen’s *d* = 0.71; see Table 3).

The observed improvement not only confirmed the algorithm’s effectiveness in enhancing anaerobic power and agility but also underscored its capability to holistically enhance cardiorespiratory function and muscular velocity. These findings further validated the superiority of personalized exercise interventions in enhancing overall physical fitness.

#### Summary

The 12-week personalized exercise intervention led to significant improvements in both BMI and multiple fitness metrics (see Table 3). These findings indicated that the algorithm, through dynamic adjustments and personalized prioritization, effectively optimizes physiological adaptation while minimizing overtraining risks. Compared with conventional exercise guidelines, this framework offers superior scientific precision and personalization by integrating HIIT, sport-specific training, and rating of perceived exertion (RPE)-based autoregulation. Notably, no serious adverse events (defined as injuries requiring medical attention) were reported, which further underscores the algorithm’s safety and efficacy. In summary, these findings validate personalized exercise interventions as an effective approach for enhancing physical fitness and supporting sustainable healthy lifestyles, while establishing an evidence-based framework for future clinical implementation.

## Discussion

The superior performance of the hybrid model stems from its dual capability, i.e., capturing local temporal patterns in lap time data through 1D-CNN architecture, and identifying global feature interactions through attention mechanisms. SHAP analysis revealed pull-up capacity as the most influential BMI predictor (mean |SHAP| = 0.18), consistent with previous studies demonstrating an inverse relationship between upper-body strength and adiposity^14^.

The effectiveness of prescription algorithm may be attributed to two factors:

1. Periodization: Microcycle adjustments based on biweekly reassessments.
2. Targeting: Development of energy system-specific interventions tailored to physiological deficits.

Notably, the prevalence of overweight decreased by 23.5%, exceeding that of comparable digital interventions (typically 8–15%)^15^, thereby highlighting the effectiveness of the AI-human hybrid coaching approach.

Furthermore, the model demonstrated robust generalization capability despite employing synthetic data augmentation. The application of SMOTE and Gaussian noise exclusively within the training folds of the cross-validation loop prevented data leakage and over-optimistic performance estimation. The final model achieved balanced precision and recall across all BMI categories (see Supplementary Table 1), and an ablation study confirmed a 12.4% improvement in macro-F1 score attributable to augmentation, without performance degradation on the pristine validation set. This indicates that the augmentation strategy effectively mitigated class imbalance while promoting the learning of invariant feature representations, rather than merely inflating performance metrics through overfitting to synthetic samples.

It is important to clarify that the current study validates the framework’s capability to generate an effective initial prescription based on a single baseline assessment. The envisioned fully adaptive system, where the model is iteratively retrained on longitudinal training response data to enable dynamic, real-time prescription refinement, represents the next phase of this research program and is beyond the scope of this validation study.

## Conclusion

This study presents a novel machine learning framework for generating personalized exercise prescriptions based on individual physical fitness profiles and BMI metrics. By integrating 1D Convolutional Neural Network (1D-CNN) with multi-head attention and Light Gradient Boosting Machine (LightGBM), the proposed architecture has demonstrated state-of-the-art performance, with an impressive accuracy of 94.5% in BMI classification. This hybrid model captures intricate temporal patterns in fitness data through 1D-CNN and attention mechanisms, while simultaneously leveraging LightGBM’s robust classification capabilities to significantly improve prediction accuracy. Its high computational efficiency, demonstrated by an inference time of less than 0.8 milliseconds per sample, underscores its suitability for real-world deployment in both clinical and mobile health settings. The integration of SHapley Additive exPlanations (SHAP) enhances the interpretability of the framework, enabling dynamic adaptation of exercise prescriptions to target specific fitness components such as muscular strength, cardiorespiratory endurance, speed, agility, and flexibility. Empirical validation through a 12-week randomized intervention trial demonstrated the clinical efficacy of the proposed approach, yielding a 23.5% reduction in overweight and obesity prevalence, alongside significant performance improvements, including a 15.2% increase in pull-up test repetitions and a 9.8% reduction in 30 × 2 shuttle run times. These findings demonstrated that the framework not only optimizes physiological adaptations but also mitigates the risks of overtraining, thereby outperforming conventional exercise prescription approaches.

Building on the methodological innovations and outcomes of the current study, we outline several prospective avenues for future research:

First, at the algorithmic level, future research may explore the integration of advanced deep learning architectures (e.g., Transformers, Generative Adversarial Networks) with the existing 1D-CNN + Attention-LightGBM framework to further enhance classification accuracy and model stability. Additionally, the incorporation of multimodal data sources (e.g., biomechanical signals, heart rate monitoring) could facilitate the development of more comprehensive and adaptive personalized exercise prescription.

Second, at the clinical application level, future studies will aim to expand participant diversity to assess the applicability of this intervention across different genders, age groups, and health conditions. Concurrently, we plan to collaborate with healthcare professionals to translate these evidence-based solutions into real-world clinical practice, providing more precise and accessible health management and exercise guidance particularly for younger populations.

Third, in terms of algorithmic optimization, reinforcement learning techniques may be leveraged to enable adaptive personalization of exercise intensity and modalities, thereby optimizing training efficacy and safety. Moreover, the explainable framework proposed in this study could be integrated with AI-driven recommendation systems to deliver more intelligent and precision-oriented physical fitness enhancement protocols.

Promising avenues for future research include the integration of wearable sensor data to enable continuous, real-time monitoring of individual physiological responses, thereby further refining personalized exercise prescriptions. Additionally, expanding the study population to encompass female and older adult cohorts would enhance the generalizability of the findings, fostering the development of more inclusive and equitable health interventions.

Building upon these multifaceted expansions and innovations, we aim to translate this research into a health intervention tool with greater practical value, thereby providing theoretical underpinnings and technical references for advancing the fields of exercise medicine and public health.

## Supplementary Information

Below is the link to the electronic supplementary material.

Supplementary Material 1

## Data Availability

The datasets generated and/or analysed during the current study are not publicly available due to institutional data governance policies and the need to protect participant privacy inthis homogeneous student cohort, but are available from the corresponding author on reasonable request.

## Declarations

### Reporting guidelines

This study adheres to the CONSORT (Consolidated Standards of Reporting Trials) checklist (for non-randomized controlled trials).
