# Supplementary material for: A machine learning framework for personalized exercise prescription based on BMI and physical fitness assessment
Source: Sci Rep. 2026 Mar 13;16:13336. doi: 10.1038/s41598-026-42405-2 (PMC13106707; doi:10.1038/s41598-026-42405-2)
Supplement: Supplementary file 1 — Supplementary Material 1 [file 41598_2026_42405_MOESM1_ESM.docx]

Supplementary Table 1. Performance metrics per BMI class for the proposed hybrid model evaluated on the unaugmented test set.

| **BMI Class** | **Precision** | **Recall** | **F1-Score** | **Support** |
| --- | --- | --- | --- | --- |
| Underweight | 0.92 | 0.89 | 0.90 | 1000 |
| Normal Weight | 0.96 | 0.97 | 0.96 | 1000 |
| Overweight | 0.93 | 0.92 | 0.92 | 1000 |
| Obese | 0.91 | 0.93 | 0.92 | 1000 |

Note: Performance metrics per BMI class for the proposed hybrid model. Metrics are averaged across the 5 folds of stratified cross-validation, evaluated solely on the original, unaugmented samples within each fold‘s test set. The Support indicates the number of samples for each class in the balanced, unaugmented evaluation set. Data augmentation (SMOTE and Gaussian noise) was applied only to the training folds.

Supplementary Table 2. Complete confusion matrix for the proposed hybrid model on the unaugmented test set.

| Actual \ Predicted | Underweight | Normal Weight | Overweight | Obese | Total |
| --- | --- | --- | --- | --- | --- |
| Underweight | 920 | 80 | 0 | 0 | 1000 |
| Normal Weight | 40 | 950 | 10 | 0 | 1000 |
| Overweight | 0 | 20 | 960 | 20 | 1000 |
| Obese | 0 | 0 | 30 | 970 | 1000 |
| Total | 960 | 1050 | 1000 | 990 | 4000 |

Note: Representative confusion matrix from one fold of the cross-validation, showing performance on the original, unaugmented test samples within that fold. The total sample count (N=4,000) and per-class distribution (1,000 each) reflect the balanced evaluation subset used for consistent performance assessment across all classes. The majority of misclassifications occur between adjacent BMI categories.
